# Response to comments on "Capnography as an aid in localizing the phrenic nerve in brachial plexus surgery. Technical note"

**DOI:** 10.1186/1749-7221-3-21

**Published:** 2008-10-22

**Authors:** Hemant Bhagat, Anil Agarwa, Manish S Sharma

**Affiliations:** 1 Department of Neuroanesthesia, All India Institute of Medical Sciences, New Delhi-110029, India; 2 Department of Neurosurgery, All India Institute of Medical Sciences, New Delhi-110029, India

## Abstract

Response to comments on 'Capnography as an aid in localizing the phrenic nerve in brachial plexus surgery. Technical note' Bhagat H, Agarwal A, Sharma MS Journal of Brachial Plexus and Peripheral Nerve Injury 2008, 3:14 (22 May 2008)

## 

Dear Editors,

We appreciate the concerns raised by the reader as they are genuine and focused. Monitoring the depth of anaesthesia with aid of specific monitors is a good idea. In our study, the doses of drugs used were according to the standard anaesthetic practice for total intravenous anaesthesia which should not warrant concerns regarding the adequacy of the depth of anaesthesia. Anaesthesia was induced with propofol 1.5–2 mg/kg and fentanyl 2 μg/kg while maintenance of anaesthesia was with propofol 6–10 mg/kg/hour. Fentanyl was administered at a dose of 1 μg/kg prior to skin incision and thence every 30 minutes to ensure adequate analgesia. The propofol infusions were adjusted according to increase in heart rate and/or blood pressure to more than 20% of baseline values. With this anaesthetic technique and use of laryngeal mask airway we were able to use controlled ventilation in all our patients. The idea of using controlled ventilation was to abolish the respiratory efforts and consequently have a uniform capnograph which would enable us to appreciate any changes in response to electrical stimulation of phrenic nerve.

Premature respiratory efforts because of inadequate anaesthesia and analgesia can cause similar pattern in the capnograph as reported by us. However we ensured adequate depth of anaesthesia and analgesia based on the haemodynamic response to surgery. The titration of anaesthesia based on haemodynamic parameters have been found to be sufficient to ensure adequate depth of anaesthesia [1]. The hemodynamic response to phrenic nerve stimulation was unremarkable.

Hiccups can occur during phrenic nerve stimulation. This is what we have exactly tried to explain. This novel technique is being described to localize the phrenic nerve in an otherwise scarred tissue. Consequently, the stimulation was attempted in and around the phrenic nerve, which may result in incomplete diaphragmatic contraction.

The ventilator rate and tidal volume were adjusted to maintain an end-tidal carbon dioxide (ETCO_2_) between 35–40 mmHg. Following electrical stimulation around the phrenic nerve with lower amplitude of electric current, there is subclinical diaphragmatic contraction (mimicking premature inspiratory efforts) and a fall in ETCO_2_. With increase in the amplitude of current, there is further increase in the force of diaphragmatic contraction (akin to hiccups) with additional fall in ETCO_2_.

We hope the response addresses the concerns of the reader.

Thanking you

Hemant Bhagat, Anil Agarwal, Manish S Sharma

## Competing interests

The authors declare that they have no competing interests. They did not receive any grants nor do they have any vested interests in the equipment described.
